# The effect of inactin on kidney mitochondrial function and production of reactive oxygen species

**DOI:** 10.1371/journal.pone.0207728

**Published:** 2018-11-26

**Authors:** Tomas A. Schiffer, Michael Christensen, Håkan Gustafsson, Fredrik Palm

**Affiliations:** 1 Department of Medical Cell Biology, Uppsala University, Uppsala, Sweden; 2 Department of Clinical Medicine, Aarhus University, Aarhus, Denmark; 3 Department of Medical and Health Sciences, Linköping University, Linköping, Sweden; University of PECS Medical School, HUNGARY

## Abstract

Inactin is a long lasting anesthetic agent commonly used in rat studies, but is also shown to exert physiological effects such as reducing renal blood flow, glomerular filtration rate and depressing tubular transport capacity. The effect of inactin on isolated kidney mitochondria is unknown and may be important when studying related topics in anaesthetized animals. The aim of this study was to determine whether inactin exerts effects on mitochondrial function and production of reactive oxygen species. Kidney mitochondrial function and production of reactive oxygen after acutely (5 min) or longer (1.5 hour) anesthetizing rats with inactin was evaluated using high-resolution respirometry. The results demonstrate that inactin significantly improves respiratory control ratio, inhibits complex I in the mitochondrial respiratory chain, reduce both unregulated proton leak and time dependently reduce the regulated proton leak via uncoupling protein-2 and adenine nucleotide translocase. Inactin also contributes to increased mitochondrial hydrogen peroxide production. In conclusion, inactin exerts persistent effects on mitochondrial function and these profound effects on mitochondrial function should to be considered when studying mitochondria isolated from animals anesthesized with inactin.

## Introduction

Anesthesia is required for most animal experiments in order to mitigate pain or distress. There are a few injectable and gaseous agents for this purpose all with pros and cons depending on applications. Gaseous applied anesthetics, such as isoflurane and halothane, are usually easy to administer and promotes rapid recovery [1]. Injectable agents, such as thiobutabarbital sodium salt hydrate (inactin), are usually long lasting anesthetics commonly used in rat studies [2–4]. Anesthesia can have significant global or local systematic impact on blood flow, oxygenation, pH, heart rate, and respiratory rate [2]. Irrespective of anesthesia used, it is important to understand the physiological effects impacted by these agents in order to correctly interpret the results. As an example, isoflurane is protective in ischemia-reperfusion via inhibition of mitochondrial permeability transition pore opening [5–7] and complex 1 in the mitochondrial electron transport system as well as via promoting increased proton flux through the ATP synthase [8]. Inactin is known to progressively lower heart rate and arterial pressure and promote hypercapnia, although to a lesser extent compared to pentobarbital [2]. Leyssac *et al*. showed that inactin depresses both resting proximal tubular transport capacity and the tubular response to a physiologically relevant volume expansion [9]. In addition, Walker and colleagues reported that administration of inactin in rats was associated with a marked reduction in renal blood flow and glomerular filtration rate [10]. Until now, it is unknown whether inactin also affects kidney mitochondrial function. Therefore, we investigated the effects of inactin on rat kidney mitochondria function using high-resolution respirometry and hydrogen peroxide production using fluorometry.

## Materials and methods

### 2.1. Chemicals

All chemicals were from Sigma-Aldrich (St Louis, MO, USA) if not otherwise stated.

### 2.2. Animals

All animal procedures were approved by the Uppsala animal ethics committee and were performed in accordance with the National Institutes of Health Guide for the Care and Use of Laboratory Animals. 12–16 weeks old male Sprague-Dawley rats were purchased from Charles River (Sulzfeldt, Germany) and given ad libitum access to tap water and pelleted standard rat chow.

### 2.3. Experimental protocol

Animals were randomly divided into control group (N = 9), acute inactin-treated group (5 min before decapitation) (N = 10) and long-term inactin-treated group (90 min) (N = 9). At the day of experiments, the control rats were decapitated and the right kidney immediately extracted and put in ice-cold mitochondrial isolation medium (sucrose 250 mM, Hepes 10 mM, EGTA 1 mM, BSA 1g/L, pH 7.4 compensated with KOH). Inactin from a stock solution dissolved in dH_2_O (0.1g/ml) was injected i.p at the concentration 120 mg kg^-1^ bw^-1^ in the treated groups. The rats in the acutely treated group (A) were decapitated approximately 5 min after the inactin injection. The rats were sacrificed and the kidneys extracted after approximately 90 min in the long-term treated group (LT). The rats in the LT group were placed at a heated operating table in order to maintain body temperature at 37.5 °C. LT rats were tracheostomized, catheters were placed in femoral artery and vein. The bladder was catheterized for drainage. Ringer solution was infused at the rate 5 ml kg^-1^ h^-1^ in order to maintain normal health status. Following the termination of the experiments, the right kidney was extracted and put in ice-cold isolation medium. The rats were under inactin induced anesthesia approximately 90 min before extraction of the kidneys. Rats with mean arterial pressure below 80 mmHg were excluded.

### 2.4. Kidney cortex mitochondrial isolation

Kidney cortex mitochondria was isolated by differential centrifugation described elsewhere [11].

### 2.5. Respirometry

Mitochondrial function was performed with high resolution respirometry (Oroboros, Innsbruck, Austria) in respiration medium containing (EGTA 0.5 mM, MgCl_2_ 3 mM, K-lactobionate 60 mM, Taurine 20 mM, KH_2_PO_4_ 10 mM, HEPES 20 mM, Sucrose 110 mM, BSA essentially fatty acid free 1 g/L). All mitochondrial substrates and various inhibitors were added to the respiration chambers using a Hamilton syringe from pre-diluted stock solutions as follows: Pyruvate (2 M in dH_2_O, freshly diluted the same day of experimentation), malate (0.8 M in dH_2_O), succinate (1 M in dH_2_O), ADP (0.5 M in dH_2_O combined with 0.3 M MgCl_2_), oligomycin (5 mM in EtOH), carboxyatractylocide (CAT; 5 mM in dH_2_O), guanosine diphosphate (GDP; 0.2 M in dH_2_O), Amplex UltraRed (Invitrogen, USA) (10 mM in DMSO) and horseradish peroxidase (Thermo Fisher Scientific, USA) (500 U/ml in mitochondrial respiration medium). Pyruvate (5 mM) and malate (2 mM) were added in order to measure state 2 respiration followed by addition of ADP (2.5 mM) to measure complex 1 mediated maximal respiratory capacity (state 3 respiration). Maximal oxidative phosphorylation capacity and succinate control ratio (SCR) was evaluated by adding succinate during complex I mediated state 3 respiration (complex I + II mediated state 3 respiration). LEAK respiration was measured in the presence of pyruvate (5 mM), malate (2 mM) and oligomycin (2.5 μM). GDP (2 mM) was added to measure uncoupling protein 2 (UCP2) dependent proton LEAK and CAT (5 μM) to measure adenine nucleotide translocase (ANT) dependent LEAK. All respiration was normalized to mitochondrial protein determined spectrophotometrically using a commercial kit (DC Protein Assay, Bio-Rad, USA). Mitochondrial oxygen affinity (p50_mito_) was determined in presence of both complex I and II substrates by allowing mitochondria respire until anoxia. The software DatLab2 (Oroboros) was used to calculate the p50_mito_. The PO_2_ range was set to < 2.5 kPa to fully cover the hyperbolic function.

### 2.6. P/O-ratio

P/O-ratio was determined by steady state infusion of ADP in order to mimic the *in vivo* situation and previously described [11]. The concentration of ADP in the infusion solution was determined spectrophotometrically with a commercially available kit (MAK081) (Merck, Germany).

### 2.7. Hydrogen peroxide production

O2k-Fluo LED2-Module (Oroboros) together with Amplex UltraRed (10 μM) (Invitrogen, USA) and horseradish peroxidase (1 U/ml) (Thermo Fisher Scientific, USA) was used to detect mitochondrial hydrogen peroxide production. Calibration was performed by adding 60 nM steps of a standard hydrogen peroxide solution (40 μM), dissolved in dH_2_O containing HCl (10 μM). H_2_O_2_ production was normalized to mitochondrial protein.

### 2.8. Statistics

One-way ANOVA followed by Tukey’s multiple comparisons test were used in order to detect significant differences between inactin-treated and untreated groups (GraphPad Prism 5.0). Data are presented as means ± SD and P<0.05 was considered significant.

## Results

### 3.1.

**Fig 1:**
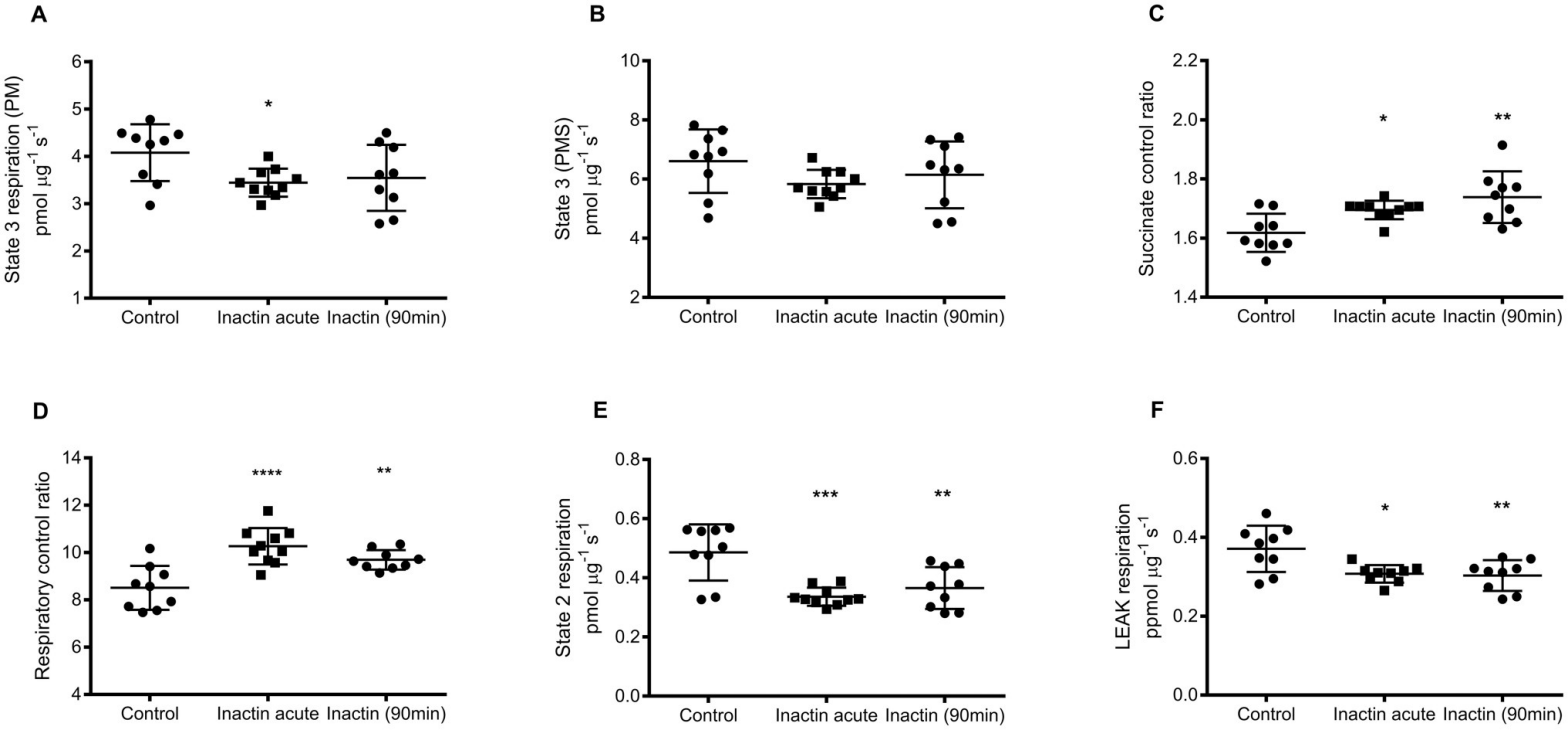
Mitochondrial respiratory function evaluated using high resolution respirometry. (A) Maximal complex I (CI) mediated OXPHOS capacity (state 3) respiration was determined in the presence of pyruvate, malate and ADP, and (B) CI + CII state 3 after addition of succinate. (C) Succinate control ratio (SCR) was calculated by dividing (CI + CII) state 3 with CI mediated state 3 respiration. (D) Respiratory control ratio was defined as state 3 respiration (pyruvate, malate) divided by state 2 respiration. (E) State 2 respiration was measured in the presence of pyruvate and malate and (F) LEAK respiration by inhibiting ATP synthase with oligomycin.

Quality of the isolated mitochondria were verified by RCR exceeded 7 in all mitochondrial preparations. Complex I mediated State 3 respiration was significantly lower in mitochondria acutely exposed to inactin (3.44±0.30 pmol μg^-1^ s^-1^; P<0.05 vs. Control) but not in mitochondria during long-term inactin exposure (3.55±0.70 pmol μg^-1^ s^-1^; P = 0.12 vs. Control) compared to Control (4.08±0.60 pmol μg^-1^ s^-1^; Fig 1A). CI + CII mediated state 3 respiration was similar in all groups (Control 6.61±1.07, acute 5.83±0.48 and long-term 6.15±1.13 pmol μg^-1^ s^-1^; Fig 1B). SCR was significantly higher in both groups exposed to inactin (acute 1.70±0.03; P<0.05 vs Control, and long-term 1.74±0.09; P<0.01 vs. Control) compared to Control (1.62±0.06; Fig 1C). RCR was also significantly higher in both groups exposed to inactin (acute 10.3±0.8; P<0.001 vs. Control), and long-term (9.7±0.4; P<0.01 vs. Control) compared to Control (8.5±0.9; Fig 1D). Also state 2 respiration was significantly lower in both groups exposed to inactin (acute 0.34±0.03; P<0.001 vs. Control, and long-term 0.37±0.07 pmol μg^-1^ s^-1^; P<0.01 vs. Control) compared to Control (0.49 ± 0.09 pmol μg^-1^ s^-1^; Fig 1E). This was also reflected by lower LEAK respiration in both groups exposed to inactin (acute 0.31±0.02; P<0.05 vs. Control, and long-term 0.30±0.04 pmol μg^-1^ s^-1^; P<0.01 vs. Control) compared to control (0.37±0.06 pmol μg^-1^ s^-1^; Fig 1F).

### 3.2.

**Fig 2:**
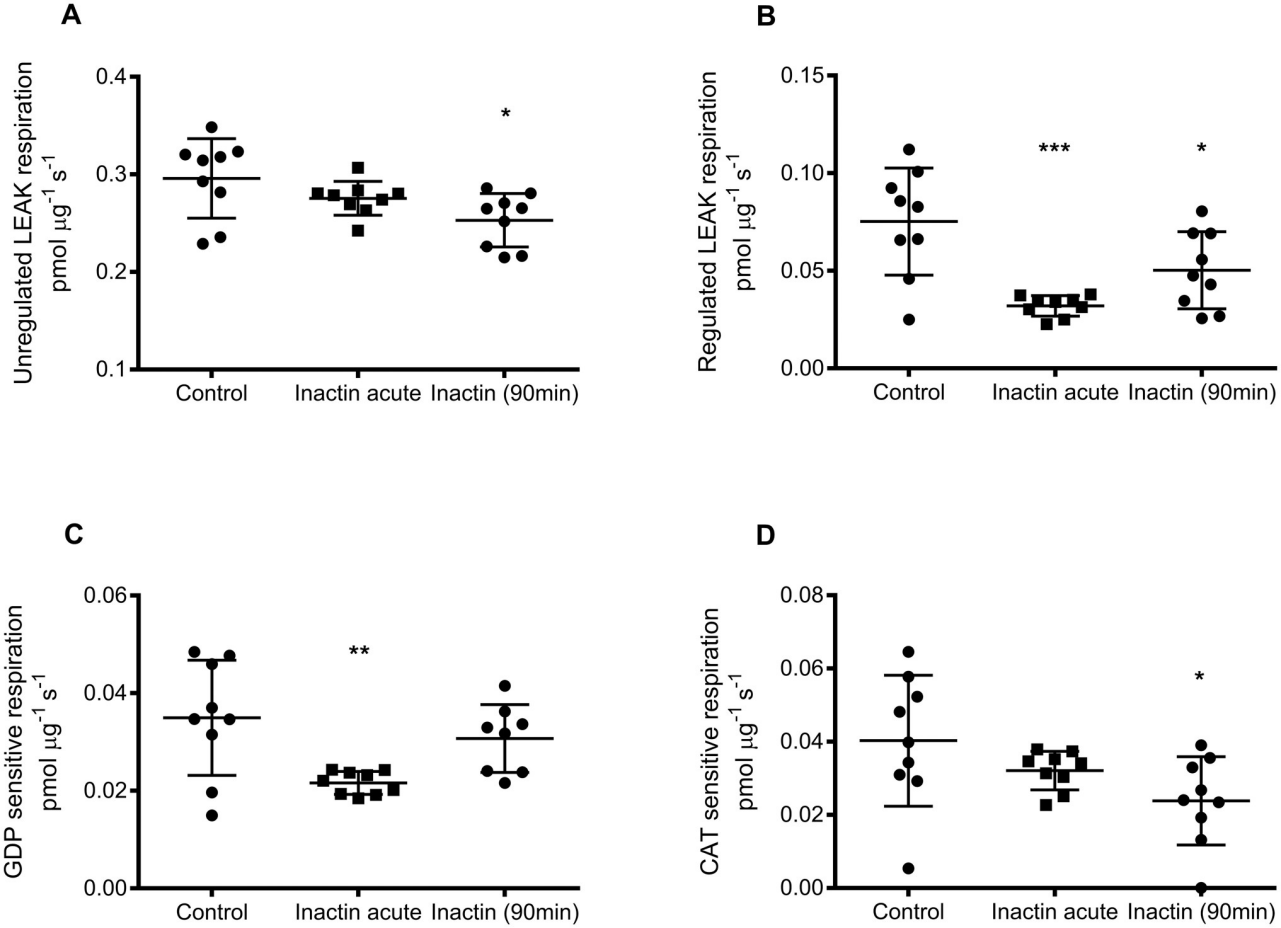
Mitochondrial LEAK respiration measured by high resolution respirometry. (A) Unregulated LEAK respiration was calculated by subtracting GDP and CAT dependent respiration from the total LEAK respiration. (B) Regulated LEAK respiration is the sum of GDP and CAT dependent respiration. (C) GDP dependent respiration was determined by adding GDP in LEAK respiration and calculating the change in respiration. (D) Similarly, CAT dependent respiration was evaluated by adding CAT during LEAK respiration.

Unregulated LEAK respiration was significantly lowered only in the group exposed to inactin long-term (0.25±0.03 pmol μg^-1^ s^-1^; P<0.05 vs. Control) compared to Control (0.30±0.04 pmol μg^-1^ s^-1^), whereas no significant effect was observed after acute exposure to inactin (0.28±0.02 pmol μg^-1^ s^-1^; P = 0.33 vs. Control; Fig 2A). Regulated LEAK respiration (GDP + CAT sensitive respiration) was also significantly lowered by inactin (acute 0.032±0.005 pmol μg^-1^ s^-1^; P<0.001 vs. Control, and long-term 0.050±0.020 pmol μg^-1^ s^-1^; P<0.05 vs. Control) compared to control (0.075±0.027 pmol μg^-1^ s^-1^; Fig 2B). Significant reduced GDP-sensitive LEAK respiration was observed in the acute inactin group (0.035±0.022 pmol μg^-1^ s^-1^; P<0.01 vs. Control), whereas GDP-sensitive LEAK respiration in the long-term inactin exposure group (0.031±0.007 pmol μg^-1^ s^-1^) was similar (P = 0.53 vs. Control) compared to control (0.021±0.002 pmol μg^-1^ s^-1^; Fig 2C). However, CAT-sensitive LEAK respiration was only affected in the long-term inactin exposure group (0.0241±0.012 pmol μg^-1^ s^-1^; P<0.05 vs. Control) whereas acute inactin exposure did not affect CAT-sensitive LEAK respiration (0.032±0.005 pmol μg^-1^ s^-1^; P = 0.39 vs. Control; Fig 2D).

### 3.3.

**Fig 3:**
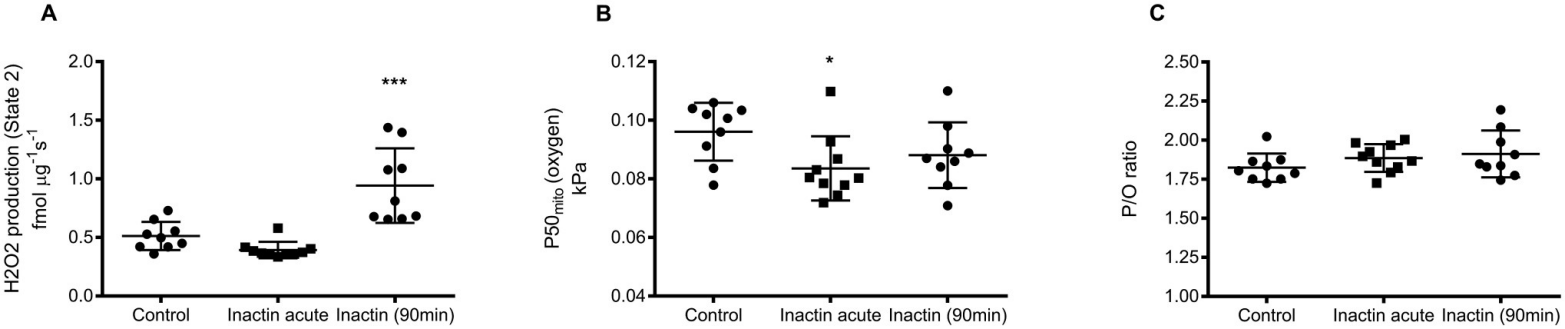
(A) Amplex UltraRed was used to spectrofluorometrically measure mitochondrial hydrogen peroxide production in CI mediated state 2 respiration. (B) Mitochondrial apparent k_M_ for oxygen (P50_mito_) was determined by allowing mitochondria respire until anoxia and calculate the hyperbolic function. (C) Mitochondrial P/O-ratio was evaluated by steady state infusion of ADP corresponding to approximately half-maximal CI mediated state 3 respiration assuming all infused ADP was converted to ATP.

 Hydrogen peroxide production during state 2 respiration was significantly increased after long-term inactin exposure (0.94±0.32 fmol μg^-1^ s^-1^; P<0.001 vs. Control) group compared to control (0.15±0.12 fmol μg^-1^ s^-1^), whereas it was unaffected by acute inactin exposure (0.39±0.07 fmol μg^-1^ s^-1^; P = 0.39 vs. Control; Fig 3A). Mitochondrial oxygen affinity (P50_mito_) was significantly decreased after acute inactin exposure (0.084±0.011 kPa; P<0.05 vs. Control) compared to control (0.096±0.010 kPa), but not significantly altered after long-term inactin exposure (0.088±0.011 kPa; P = 0.27 vs. Control; Fig 3B). Inactin had no significant effect on P/O-ratio in any of the groups (Fig 3C).

## Discussion

The present study shows that inactin exerts persistent effects on mitochondria that evidently outlast the isolation protocol. The lower C1 mediated State 3 respiration after acute exposure suggests that inactin inhibits complex 1, further supported by the higher SCR in both groups exposed to inactin. This effect is similar to what previously has been reported for isoflurane [8].

The increased RCR in combination with reduced state 2 and LEAK respiration indicate that inactin inhibits proton conductance across the inner membrane. This proton leak consists of both a regulated and an unregulated part, both reduced by inactin. The precise mechanism for unregulated proton leak is not fully understood, but it is generally not attributed to mere biophysical leak across the phospholipid bilayer [12]. The composition of cardiolipin in the mitochondrial membrane has garnered some attention due to the correlation between fatty acyl composition and mitochondrial proton leak [13]. However, such correlation cannot be observed when measuring proton conductance in liposomes derived from mitochondria with different phospholipid composition [14]. Liposomes, however, show extreme phospholipid asymmetry with only about 7% of the cardiolipin present in the outer leaflet of the liposomes [15]. Hoch and colleagues presented a model in which cardiolipin may work as an antenna by attracting protons and therefore plays and role in the unspecific proton conductance [16]. It has also been speculated that basal proton leak may be a general property of most mitochondrial carrier proteins [17]. Identification of the exact molecular mechanism affected by inactin on unregulated proton leak is beyond the scope of this paper. The reason for the time-dependent reduction of unregulated proton leak might relate to the pharmacokinetic properties of inactin. The regulated part of mitochondrial proton leak in kidneys consists of UCP2 (GDP sensitive) and adenine nucleotide translocase (ANT) (CAT sensitive) dependent proton leak. UCP2 is activated by superoxide in the presence of fatty acids [10] and reactive alkenals, such as 4-hydroxynonenal [18], whereas ANT is activated by fatty acids and reactive alkenals [18]. Interestingly, acute inactin exposure inhibited proton leak mainly through UCP2 whereas long-term exposure inhibited proton leak via ANT. A possible explanation would be an acute inactin mediated reduction in ROS production. This is contradicted by the unaffected hydrogen peroxide production after acute exposure to inactin. It is however not completely established whether hydrogen peroxide production fully reflects the superoxide production, which is the main reactive oxygen species produced during mitochondrial respiration. A recent paper demonstrated that complex I contributes to both hydrogen peroxide and superoxide production at two different sites with NADH-dependent change in the ratio between the production of these radicals [19]. In addition, we have noted a lack of correlation between production of superoxide radicals and hydrogen peroxide when measuring superoxide radical production using electron paramagnetic resonance with spin probes and hydrogen peroxide production using Amplex Red (unpublished). Therefore, the effects of inactin on mitochondrial superoxide production after acute inactin exposure still remains uncertain even though hydrogen peroxide production was unchanged in the current study. Inactin-mediated conformational changes of UCP2 contributing to reduced proton leak is also a possibility. However, this is contradicted by the restored UCP2-dependent leak in the long-term exposed animals. The restoration of UCP2 activity after long-term exposure to inactin occurs in parallel to increased hydrogen peroxide production.

The observed stepwise reduction of ANT-mediated proton leak may be explained by a direct interaction between ANT and inactin in relation to the pharmacokinetic properties of inactin. Another possibility is that the interface of the lipid bilayer and ANT may have been affected and therefore reduces proton leak [17]. This is somewhat supported by the correlation between the unregulated and ANT-dependent proton leak when including all groups (R^2^ = 0.23, P = 0.01) (Figure not shown).

Reduced proton leak across the inner mitochondrial membrane promotes increased membrane potential resulting in a reduction of the electron transport system, which favors radical production [20]. Indeed, hydrogen peroxide production during state 2 respiration was significantly increased by inactin. Mitochondrial oxygen affinity, defined as oxygen tension where maximal mitochondrial respiration is inhibited by 50% (p50_mito_), is dependent on proton leak and the relative activities of the electron transport system complexes [21]. The lower p50_mito_ after acute inactin opposes the effect of increased proton leak on p50_mito_ [21]. The explanation for the inactin dependent lower p50_mito_ is therefore likely related to the observed complex I inhibition, which alters the relative activity between complex I and complex IV.

A possible limitation of the study potential mitochondrial effects due to the additional surgical procedures, such as tracheostomy and cathetherization, in the long-term inactin exposure group. Although the surgical procedures may have had unknown effect on mitochondria function, the main findings in the acutely treated group are in line with the long-term treated group, implying that it is inactin that is causing the observed mitochondria- effects.

In conclusion, the present study demonstrate that inactin has several acute and long lasting effects on mitochondria function. These effects include inhibition of complex I of the electron transport chain, reduced proton leak across the inner mitochondrial membrane and increased production of reactive oxygen species. These significant effects should be considered when performing studies on isolated mitochondria, or processes influenced by mitochondrial function, in experimental setups using inactin.
